# The neural mediators of kindness-based meditation: a theoretical model

**DOI:** 10.3389/fpsyg.2015.00109

**Published:** 2015-02-12

**Authors:** Jennifer S. Mascaro, Alana Darcher, Lobsang T. Negi, Charles L. Raison

**Affiliations:** ^1^Department of Anthropology, Emory UniversityAtlanta, GA, USA; ^2^Center for Translational Social Neuroscience, Emory UniversityAtlanta, GA, USA; ^3^Department of Religion, Emory CollegeAtlanta, GA, USA; ^4^Department of Psychiatry, College of Medicine, University of ArizonaTucson, AZ, USA; ^5^The John and Doris Norton School of Family and Consumer Sciences, College of Agriculture and Life SciencesTucson, AZ, USA

**Keywords:** empathy, compassion, meditation, compassion meditation, loving-kindness meditation, oxytocin, simulation, mentalizing

## Abstract

Although kindness-based contemplative practices are increasingly employed by clinicians and cognitive researchers to enhance prosocial emotions, social cognitive skills, and well-being, and as a tool to understand the basic workings of the social mind, we lack a coherent theoretical model with which to test the mechanisms by which kindness-based meditation may alter the brain and body. Here, we link contemplative accounts of compassion and loving-kindness practices with research from social cognitive neuroscience and social psychology to generate predictions about how diverse practices may alter brain structure and function and related aspects of social cognition. Contingent on the nuances of the practice, kindness-based meditation may enhance the neural systems related to faster and more basic perceptual or motor simulation processes, simulation of another’s affective body state, slower and higher-level perspective-taking, modulatory processes such as emotion regulation and self/other discrimination, and combinations thereof. This theoretical model will be discussed alongside best practices for testing such a model and potential implications and applications of future work.

## INTRODUCTION

Over the last 25 years, research on meditation has advanced in domains both clinical and basic, motivated by an often implicit conviction that mindfulness and attention practices are effective interventions for remediating psychopathology and augmenting well-being and resilience, and may be used as tools to help scientists understand the human brain, body, and brain–body connections. More recently, researchers have turned their attention to kindness-based practices, frequently in search of answers to the dual questions of, “Can kindness be trained?” and “Are kindness-based practices good for us?” Increasingly, the answer to both of these questions appears to be yes.

There is a growing body of research on the effects and efficacy of kindness-based contemplative practices including compassion (CM) and loving-kindness (LKM) meditation [reviewed in Hofmann et al. (2011) and Galante et al. (2014)], a handful of which are studies exploring their effects on neural structure and function (Lutz et al., 2008a; Desbordes et al., 2012; Klimecki et al., 2013b,c; Mascaro et al., 2013b; Weng et al., 2013; Garrison et al., 2014). However, this sub-field remains in its infancy, and missing from this research are coherent theoretical models with which to test the mechanisms by which these meditation practices may alter the brain and body. We believe such models have dramatically increased the rigor of mindfulness research (Shapiro et al., 2006; Hölzel et al., 2011; Vago and Silbersweig, 2012), and what follows is meant as a first contribution toward building such a dialog for scaffolding future research on compassion and loving-kindness meditation.

A crucial starting place for such a model of the impact of CM and LKM on social cognition and neurobiology is with clear definitions and descriptions of both the contemplative practices in our focus and the social cognitive skills and traits in question, (for discussions of the importance of accurate construct definition, see Lutz et al., 2008b; Batson, 2009). In his review of historical trends surrounding the study of empathy, Davis (1996, p. 11) observed that “the study of empathy, as much as any topic in psychology, has been marked by a failure to agree on the nature of and relations among its core constructs.” As a result of ongoing vacillations in the importance assigned to either cognitive or affective factors by researchers in the field, the confusion noted by Davis has diminished only slightly since the time of his writing (Batson, 2009; see **Table 1** for the relationship between terms used here and related terms). While not in complete agreement, social cognitive neuroscientists and social psychologists generally converge on a definition of *empathy* as an affective response that arises from the comprehension of another’s emotional state and that is similar to what the other person is feeling (Eisenberg et al., 1991; de Vignemont and Singer, 2006). More recently, social cognitive neuroscientists have turned their attention to the related but arguably distinct construct, *compassion*, usually defined as the deep wish that another be free from suffering, coupled with the motivation to alleviate such suffering (Kim et al., 2009; Klimecki et al., 2013b). It is generally agreed that empathy and/or compassion can lead to *prosocial behavior* or *altruism*, helping behavior directed at another in need or distress (De Waal, 2008).

**Table 1:** Associations between terms used here and related terms used in social cognitive neuroscience and social psychology (Preston and De Waal, 2002; Keysers and Gazzola, 2007; De Waal, 2008; Singer and Lamm, 2009; Preston and Hofelich, 2012; Zaki and Ochsner, 2012).

Current terms	Related terms
Perceptual/motor	Mirror simulation
Affective	Simulation Resonance Emotional contagion
Cognitive	Perspective-taking Theory of mind Mentalizing
Compassion	Sympathy Prosocial concern Empathic concern
Prosocial behavior	Altruism Empathic motivation

In what follows, we will start with a brief treatment of traditional contemplative accounts of empathy and compassion found in Buddhist traditions, as well as a description of the primary meditation practices currently undergoing scientific scrutiny. Next, we will outline a theoretical model arising from current research in social psychology and social cognitive neuroscience, which proposes core neural components of empathy, compassion, and prosocial behavior, coupled with testable hypotheses regarding how compassion practices may alter these components. Finally, we will situate existing neurobiological studies within this testable model and end with a discussion of best practices for investigating the mechanisms of compassion and for targeting populations that may benefit from compassion and loving-kindness meditation.

## CONTEMPLATIVE ACCOUNTS AND PRACTICES

With a relative torrent of recent research on the neurobiology supporting empathy, it is striking that social cognitive neuroscientists have only recently come to appreciate the distinction between empathy and compassion, with this development arising largely from its interaction with Buddhist contemplatives (for example, Davidson et al., 2002). According to the Indo-Tibetan Buddhist tradition, compassion is based upon the fundamental appreciation of interdependence and the illusory nature of the self (Wallace, 2001). Here, the granularity with which this contemplative tradition characterizes positive emotions is striking, as compassion is cultivated along with three other discrete qualities (loving-kindness, empathetic joy, and equanimity), which are together referred to as the *four immeasurables*. Loving-kindness, translated from the Pali term, *mettā*, is defined as the wish that others find genuine happiness and well-being. While empathy involves taking another’s perspective in order to experience their emotional state and is a foundation of compassion, compassion is the wish that others be free from suffering (Wallace, 2001).

Our understanding of compassion and empathy has also been enriched by phenomenological accounts from contemplative adepts such as Matthieu Ricard. A renowned student and practitioner of the Nyingma school of Tibetan Buddhism, Ricard describes two distinct and refined states under his command during meditation practice: “So when I was immersing myself in empathic resonance, I visualized the suffering of these orphan children as vividly as possible. The empathic sharing of their pain very quickly became intolerable to me and I felt emotionally exhausted, very similar to being burned out... Subsequently engaging in compassion meditation completely altered my mental landscape. Although the images of the suffering children were still as vivid as before, they no longer induced distress. Instead, I felt natural and boundless love for these children and the courage to approach and console them” [in Klimecki et al. (2013a, p. 279)].

In addition to these Buddhist theoretical models and phenomenological accounts from contemporary Buddhist adepts, Buddhist texts are rich with practices offered for enhancing the four immeasurables. For example, the Tibetan practice of *tonglen* (“giving and taking”) involves visualizing ‘giving’ one’s joy and happiness to others as an expression of love and kindness, and ‘taking’ upon one’s self the suffering of others to deepen one’s compassion (Wallace, 2001). Another set of practices described by the Indian Buddhist monk, Shantideva, involves first meditating on the *equality* of self and other, with the goal of cultivating a cherishing attitude for one’s self and others in an equal degree. Next, practitioners *exchange* their priorities to give preference to others’ interests over one’s own (Thompson, 2001; Tsong-Kha-Pa, 2004).

Currently, the most researched Buddhist kindness-based contemplative practice is Loving-Kindness Meditation. According to this practice, loving-kindness is first generated for oneself in order to remove negative emotions that might impede the generation of loving-kindness for others. Next, practitioners generate feelings of loving-kindness for someone whom it is typically easy, for example, someone who is acutely suffering or a close loved one. The practitioner progresses by extending this feeling to others for whom loving-kindness may be more challenging, first, to someone neutral, and ultimately to someone whom is challenging or difficult (Wallace, 2001; Salzberg, 2002). Currently, there are several adapted versions of LKM under investigation (Fredrickson et al., 2008; Johnson et al., 2011; Jazaieri et al., 2013, 2014).

A second practice currently being examined is Cognitively-Based Compassion Training (CBCT), based on the 11th century Tibetan Buddhist *lojong* (“mind training”) tradition and heavily rooted in the seminal works of eight century Buddhist adept Shantideva. In its operationalization for novice populations, CBCT modifies standard *lojong* procedures in two important ways. First, the program is presented in a secular manner; thus, all discussions of soteriological or existential themes (e.g., the attainment of Buddhahood, Karma) are omitted. Second, rather than commencing with compassion-specific techniques, CBCT provides an introduction to foundational meditation practices; specifically, 1 week each of concentrative (i.e., *shamatha*) and open-presence practices at the beginning of the course. While these techniques are generally considered advanced according to the Tibetan tradition, they are often practiced alongside compassion practices and are thought to be necessary for establishing the focus and awareness necessary to engage in analytical practices (HHDL, 2001; Wallace, 2001). In contrast to the affective focus of LKM, CBCT uses analytical and didactic techniques to reorient the practitioner’s perspective on his or her relationship with others. It is through this active analytical process and reorientation that empathy and compassion are cultivated (Ozawa-de Silva and Dodson-Lavelle, 2011). The instruction unfolds in the following order:

**Module 1:** Developing Attention and Stability of Mind

**Module 2:** Cultivating Insight into the Nature of Mental Experience

**Module 3:** Cultivating Self-Compassion

**Module 4:** Developing Equanimity

**Module 5:** Developing Appreciation and Gratitude for Others

**Module 6:** Developing Affection and Empathy

**Module 7:** Realizing Wishing and Aspirational Compassion

**Module 8:** Realizing Active Compassion for Others

Another contemplative program that incorporates analytic strategies is compassionate mind training (CMT) and its more encompassing psychotherapeutic application, Compassion-Focused Therapy (CFT; Gilbert, 2009). A clinically informed practice constructed as a therapeutic tool, CFT incorporates a Buddhist understanding of compassion alongside the cultivation of emotion regulation skills and the augmentation of secure attachment (Gilbert, 2013), with the idea that by instilling feelings of safety and decreasing negative emotions, the patient will grow their compassion, and in turn, their well-being (Gilbert, 2014a). We characterize CFT as an analytical practice for the purposes of this review given its connections with cognitive behavioral therapy and its use of reason and imagery to generate awareness in the practitioner of the importance of being a “compassionate self” (Gilbert, 2014b). However, it is important to note that CFT incorporates a wide array of practices to maximize its therapeutic potential (for a thorough description, see Gilbert, 2014b). Interestingly, CFT entrusts the therapist to model the components of compassion in a way that imparts those skills on their patient (Gilbert, 2009), and an intriguing hypothesis is that the therapist benefits alongside the patient. While several studies attest to the efficacy of CFT (Gale et al., 2014; Heriot-Maitland et al., 2014), the neural mediators, to the best of our knowledge, have remained unexplored.

Research on CBCT, LKM, and CFT/CMT presents an important opportunity not only to investigate the efficacy of the practices for enhancing well-being and prosocial concern, but also to examine whether the practices have differential effects on the brain, body, and behavior. Such research would improve our understanding of the active ingredients in each practice at the same time that it would prove a powerful tool for testing basic scientific models such as the one presented below. It is plausible that LKM explicitly targets the more affective components of empathy, while CBCT impacts the more cognitive components, and CFT may combine the effects of both LKM and CBCT.

Intriguingly, the aforementioned practices also have a common foundational thread, which is a fundamental realization that empathy and compassion are malleable and can be cultivated and optimized. In fact, while the third section of CBCT, *Cultivating Self-Compassion*, can be easily misunderstood as something akin to self-esteem, the teachings and practices are in actuality designed to help practitioners reflect on their innate ability to cultivate and shape their mind. Importantly, a recent line of research shows that individual differences in the belief that empathy can be shaped and developed predicts an individual’s propensity to empathize in difficult situations (Schumann et al., 2014). This research suggests that one of the active ingredients in compassion meditation may be simply, but repeatedly, empowering practitioners with the understanding that empathy and compassion are traits that can be cultivated. If this is the case, we would expect to see similar effects on neural systems regardless of the practice, just as we might find that compassion meditation has a similar effect to other experimental inductions or interventions that engender beliefs in the malleability of empathy and compassion. A second prediction would be that the effects of all kindness-based practices would be most pronounced during situations when empathy is most challenging, and might lead to a positive feedback whereby new empathy “successes” reinforce the practitioners’ confidence that their compassionate “muscles” are, in fact, malleable.

## NEUROSCIENTIFIC ACCOUNT

Following the western scientific definition of empathy above, an empathic response is thought to have two crucial constituents: (1) an affective dimension that involves a shared affective experience, and (2) a cognitive dimension that includes the ability to understand or have some degree of conscious awareness that the affective experience is evoked by another. If either constituent is missing, the feeling becomes something else entirely. Should the cognitive piece be missing, the observer is instead experiencing *emotional contagion* or *simulation*. Should the affective dimension be absent, the observer is using their *theory of mind* or *perspective-taking* skills. Together these constituents combine to form a fully empathic response (de Vignemont and Singer, 2006; Eisenberg and Eggum, 2009).

**FIGURE 1:**
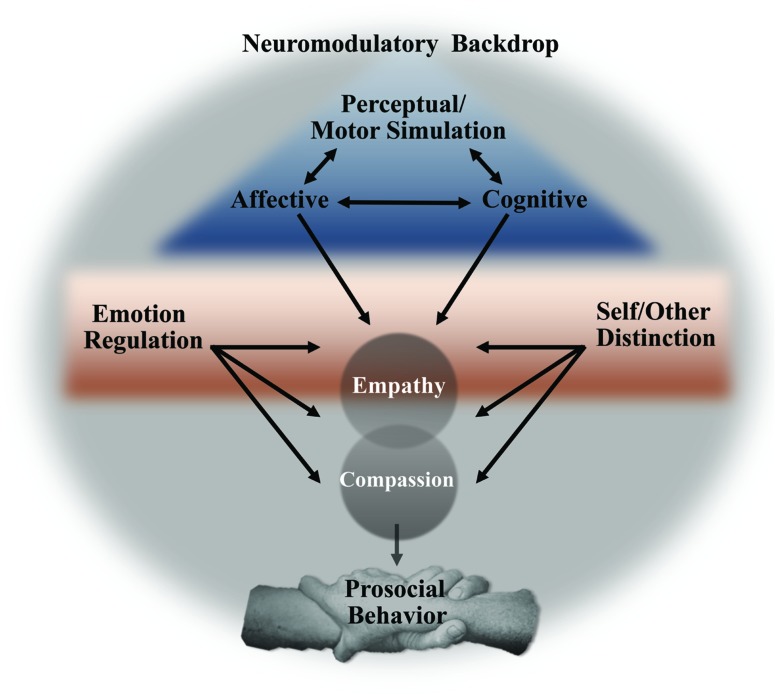
**Proposed model linking core neural processes, active amidst a neuromodulatory backdrop, leading to empathy, compassion, and prosocial behavior**.

Synthesizing almost a decade of functional neuroimaging research into a mechanistic model (**Figure 1**), it appears that three components underlie the neural bases of empathy: early and fast perceptual and motor simulation processes, affective simulation, and slower, cognitive processing. In addition to these core processes, empathy may require a self-other distinction and emotion regulation. All of these processes take place in, and are influenced by, a neuromodulatory milieu that, as we will see, takes cues from the environment and may serve as a powerful target for contemplative practices. Each of these three levels of neural influence and their possible control by meditation practices will be elaborated, but it is important to remember that this model is offered for heuristic purposes, with the acknowledgment that these processes and neural systems are multifaceted and likely influence one another in complex ways that are yet undiscovered. For example, several researchers have noted the important distinction between *capacity* and *propensity* when it comes to empathy (Klein and Hodges, 2001; Keysers and Gazzola, 2014), and it is likely that feelings of compassion alter an individual’s propensity to empathize. It should also be noted, as many have before, that prosocial behavior does not necessarily rely on each or even any of these components (De Waal, 2008; Preston and Hofelich, 2012; Decety and Cowell, 2014), just as compassion can likely occur in the absence of empathy, as will be discussed in more detail below. With these caveats in mind, we will detail the neural systems that contribute to empathy and compassion.

### PERCEPTUAL/MOTOR

Though not consistently activated by many of the empathy-for-pain tasks utilized by functional neuroimagers (Fan et al., 2011; Lamm et al., 2011), the amygdala is arguably a core structure that subserves empathy and compassion. The first evidence supporting its importance for empathy came from studies of psychopaths, whose deficits in empathy form a core symptom of their disorder and who consistently have altered amygdala structure and function (Rilling et al., 2007; Blair, 2008; Marsh et al., 2013). Beyond its role in the etiology of psychopathy, recent studies also support the amygdala’s role in empathy in healthy populations. For example, a recent study found that extreme altruists have greater amygdala volume and activity when viewing others’ distressed faces (Marsh et al., 2014), and another study found that individuals that self-report high levels of affective empathy have greater functional connectivity between the amygdala and other limbic structures consistently implicated in empathic processing [anterior insula (AI); Cox et al., 2012].

However, the amygdala’s implication in empathy rests in large part on correlational studies such as those referenced above [though see (Leigh et al., 2013) for the effects of acute amygdala lesion on affective empathy] and its exact role remains unclear. Some have argued that the importance of the amygdala in this context stems from its role in detecting the salience of, and learning about, social information based on sensory cues (Blair, 2008), which may be critically involved in the affective dimension of empathy (Hurlemann et al., 2010). For example, the amygdala plays a crucial role in detecting social information from others’ eyes (Mosher et al., 2014) and in emotional processing of visual information (Pessoa and Adolphs, 2010; Wang et al., 2014), and it is well-placed to translate incoming sensory information into changes in arousal (Davis, 1992). It is possible that kindness-based meditation practices alter these early perceptual processes to direct an observer’s attention and resources toward a target that is suffering.

A second early system that is often implicated in empathy is the putative ‘mirror neuron system,’ composed of the anterior part of the inferior parietal lobe and the inferior frontal cortex (Iacoboni and Dapretto, 2006). This system is thought to facilitate emotional understanding by mapping the target’s emotive facial expression onto the observer’s premotor repertoire. As such, neural activity related to motor simulation supports the ability to read emotional facial expressions (Carr et al., 2003; Jabbi and Keysers, 2008), and there is evidence that activity in this system precedes and may be causal to activity in the affective system described below (Jabbi and Keysers, 2008). In our longitudinal investigation of CBCT, we found that those randomized to meditation, compared to a health education control group, had enhanced scores on an empathic accuracy task. Increased scores were related to increased activity in the inferior frontal gyrus, a hub in the putative mirror neuron system, and the dorsomedial prefrontal cortex, a region that we will see below is important for thinking about others’ mental states (Mascaro et al., 2012).

### AFFECTIVE

A second component of empathy is often referred to as affective simulation, a process of matching limbic system activity with that of the target. Consistently, both the perception (auditory and visual) and contemplation of the suffering of another elicits activation in the anterior mid-cingulate cortex (aMCC), as well as bilateral AI and ventral frontal operculum, particularly on the right side (Lamm et al., 2011). Activity in the AI is thought to represent a simulated mapping of the observed individual’s body state onto one’s own (Fan et al., 2011; Bernhardt and Singer, 2012). Two studies have linked subsequent prosocial behavior with AI activity when viewing another’s suffering (Hein et al., 2010; Masten et al., 2011). Importantly, these results were found using different paradigms, with one study inducing empathy in subjects by leading them to believe others were being excluded in a ball-tossing game (Masten et al., 2011) and the other had subjects watch others receive painful shocks and then gave them the choice to endure painful shocks on behalf of the other (Hein et al., 2010). In both cases, the finding that altruistic behavior was predicted by AI activity supports the idea that affective simulation is, at least in some cases, causal to compassion and prosocial behavior.

### COGNITIVE

The third component of empathy is the cognitive element, often referred to as perspective-taking or mentalizing, which allows the observer to at some level understand that his or her affective state is related to someone else’s affective state. Mentalizing consistently activates the medial and dorsomedial prefrontal cortex and the temporoparietal junction (TPJ), systems that are thought to subserve relatively controlled, reflective cognition (Lieberman, 2007). These neural regions are also activated by a diverse array of empathy-inducing tasks (Lamm et al., 2011; Morelli et al., 2012).

Given the analytical nature of CBCT, it is worth speculating that training augments regions of the brain important for mentalizing. Consistent with this, our longitudinal study found that enhanced empathic accuracy scores were in part related to enhanced activity in the dorsomedial PFC (Mascaro et al., 2012). An intriguing hypothesis is that these results reflect early effects of CBCT, and that with more extensive practice would come changes in the affective and motivational systems thought to subserve compassion, and described more fully below.

### EMOTION REGULATION

Research from social and developmental psychology has convincingly demonstrated a difference, both in subjective feeling and in resultant behavior, between *empathy* and the related but distinct experience of *personal distress* (Batson et al., 1983; Eisenberg et al., 1998). Batson explains personal distress: “This state does not involve feeling distressed *for* the other or distress *as* the other. It involves feeling distressed *by* the state of the other.” (Batson, 2009) As evidence, cross-cultural studies (Germany, Israel, Indonesia, and Malaysia) in preschool aged children consistently reveal a positive relationship between empathy (e.g., child shows features of sadness and has a soft voice toward an experimenter whose balloon had popped) and prosocial helping behavior. However, there was a negative relationship between self-focused distress (child turns away from victim, interpreted as avoidance of the distressing stimuli) and prosocial behavior (Trommsdorff et al., 2007). Interestingly, Buddhist contemplative accounts are consistent with this idea:

“When one empathetically attends to another person who is unhappy, one naturally experiences sadness oneself. But such a feeling may actually lead instead to righteous indignation and the vengeful wish to exact retribution on the one who has made the other person unhappy. On the other hand, in the cultivation of compassion, empathetic sadness or grief acts instead as fuel for the warmth of compassion. One does not simply remain in a state of sadness or despair, but rises from this with the wish: ‘May you be free of this suffering and its causes!”’ (Wallace, 2001, pp. 11–12)

Taken together, these data suggest that becoming mired in personal distress is distinct from empathy and impairs prosocial behavior. It is likely, then, that emotion regulation plays an integral role in determining an individual’s response to viewing another’s suffering.

Defined as the initiation of new, or modulation of ongoing, emotional responses, emotion regulation varies in method and speed of processing from changes in attention to more cognitive reappraisal strategies (Ochsner and Gross, 2005). For example, simply shifting attention toward or away from social cues can up- or down-regulate empathic processes (Zaki, 2014), a regulatory process that arguably involves the amygdala, in some cases relying on it (Todd et al., 2012), in other cases modulating it (Larson et al., 2013). One testable hypothesis is that individuals motivated toward a compassionate response by meditation modulate their attention toward a suffering other in such a way to hover in a sweet spot, empathic but not over-aroused.

In addition to attention-shifting, cognitive reappraisal may modulate empathy by altering emotional responding. Zaki (2014) presents a detailed model of empathy-specific appraisals that are influenced by approach and avoidance motivations to determine empathy across contexts. In general, cognitive strategies activate the lateral (Ochsner et al., 2002) and ventromedial (Urry et al., 2006) prefrontal cortex. Interestingly, cognitive reappraisal strategies involving prefrontal regions are generally linked with *reduced* activation of the amygdala (e.g., Banks et al., 2007), suggesting that, while cognitive reappraisal is certainly not mutually exclusive with attention-shifting, the two different types of emotion regulation may have differing functional profiles in the amygdala. In addition to the prefrontal cortex, cognitive reappraisal strategies also engage the vagus nerve’s parasympathetic influence over heart rate, as reflected by respiratory sinus arrhythmia (RSA; Butler et al., 2006; Segerstrom and Nes, 2007). Porges’s (2003, 2007) Polyvagal theory posits that it is this vagal brake, shaped by evolutionary pressures for parental caregiving, that supports affiliative interactions. Researchers have found that compassionate responses appear to rely on parasympathetic dampening of the emotional response of witnessing another’s suffering (Rockliff et al., 2008).

While emotion regulation is often hypothesized as an integral outcome of mindfulness meditation (e.g., Hölzel et al., 2011), few studies, to our knowledge, have directly investigated whether kindness-based meditation practices augment emotion regulation. However, a recent study found that LKM increased practitioners’ vagal tone, an effect that moderated an increase in positive emotions, which in turn moderated even greater gains in vagal tone (Kok et al., 2013). Importantly, the positive spiral of increased vagal tone was mediated by increased feelings of social connectivity. While the researchers assessed vagal tone at rest, an interesting next step would be to examine whether these gains in vagal tone are evident during an empathy-inducing situation. Weng et al. (2013) randomized subjects to either 2 weeks of LKM or to a control course that taught emotional reappraisal strategies. Those randomized to LKM had increased neural activity while viewing photographs of others suffering in an area of the putative mirror-neuron system (inferior parietal lobe) and in a brain region important for emotion regulation [dorsolateral PFC (dlPFC)], and they exhibited more altruism during an economic game outside of the scanner. Functional connectivity between the dlPFC and the nucleus accumbens (NA) predicted greater altruistic behavior, a finding the authors interpreted as consistent with the idea that LKM enhances altruism by augmenting emotion regulation in the face of suffering.

Should kindness-based meditation augment prosocial emotions and behavior by enhancing emotion regulation and vagal tone in response to others’ suffering, there may be mediating mechanisms in addition to the enhanced feelings of connectivity reported by Kok et al. (2013). For example, van Kleef et al. (2008) found that individuals who self-report higher-levels of social power exhibit less vagal tone and compassion in response to another’s suffering, and it may be that compassion meditation alters feelings of social power by reminding practitioners of their interdependence and shared desire for happiness with others.

### SELF/OTHER DISTINCTION

Nearly two decades of research from social psychology shows that excessive overlap between self and other may render the perceiver mired in personally oriented distress that, rather than leading to prosocial behavior, leads to disengagement from the victim (Batson et al., 1987; Batson, 1998). In addition to this cross-sectional research, Hoffman (2001) cites developmental research in support of the same idea. While young children display “egocentric empathic distress” causing them to seek personal comfort when they witness another in distress (for example, by crawling into their parent’s lap), the development of a self-concept is concomitant with a child’s tendency to make helpful advances toward the victim (Hoffman, 2001). Several studies have found that mirror-self recognition in children predicts later helping behavior during empathic distress (Zahn-Waxler et al., 1979; Johnson, 1982; Bischoff-Kohler, 1991). Based on these data, social cognitive neuroscientists have persuasively argued for the importance for empathy of a rigid self/other distinction (Decety and Grèzes, 2006), and experimental induction of a self-oriented versus other-oriented perspective reveals that taking the perspective of another who is suffering activates the posterior cingulate cortex and TPJ (Jackson et al., 2006).

Interestingly, the importance of a self/other distinction for empathy and compassion may be one topic where current neuroscientific theories differ from contemplative accounts that emphasize the importance of self/other exchange (Thompson, 2001; Wallace, 2001). To the best of our knowledge kindness-based meditation practices have not been shown to impact the TPJ or to increase the ability to take an other-oriented perspective; however, a recent study by Garrison et al. (2014) may lend support to the idea that loving-kindness meditation reduces self-oriented processing. In this study, experienced meditators practicing LKM in the fMRI scanner had reduced functional connectivity between nodes of the default mode network thought to be important for self-referential processing (Garrison et al., 2014).

### COMPASSION

Clarifying the distinction between compassion and empathy may be highlighted as an example of the promise of functional neuroimaging, as recent studies of these discrete affective states reveal distinctly different patterns of brain activation. In fact, one of the first neuroimaging studies that purported to probe the neural correlates of compassion likely evoked empathy, and as such, the neural response to the empathy-inducing stimuli was characteristic of the core network described above (Immordino-Yang et al., 2009). However, Kim et al. (2009) found that adopting a true compassionate stance when viewing photographs of others suffering activated the mesolimbic dopamine (DA) system [ventral tegmental area (VTA) and ventral striatum] implicated in reward and motivation. A more recent study found that activity in the septal nuclei, another area important for reward and motivation, was commonly activated by several different empathy-inducing tasks and predicted helping behaviors (Morelli et al., 2012).

Interestingly, the research on compassion dovetails with that emerging from the investigation of the neurobiology of the parental brain. Animal models have long implicated both the septal area (Francis et al., 2000) and the DA system in supporting the motivation to proactively nurture offspring, with DA-producing cell bodies in the VTA projecting to the NA to motivate caregiving (Numan and Stolzenberg, 2009). Recent neuroimaging research suggests that this system may support human parents’ motivation to nurture their offspring (Mascaro et al., 2013a; Rilling, 2013), which raises the intriguing possibility that it is this system that underlies the motivational quality of compassion (Preston and Hofelich, 2012).

In fact, there is accumulating evidence that LKM alters the reward and motivation system in ways that support compassion. Klimecki et al. (2013b) found that 1 day of training in a loving-kindness practice enhanced neural responses to viewing video vignettes of others suffering in key nodes of the DA system (VTA and orbitofrontal cortex) and augmented self-reported positive affect. In a second study, the same group compared changes in the neural response to the same vignettes and found differential effects of training depending on whether the individual was trained to share others’ suffering (empathy) or in loving-kindness training. After the former, participants had enhanced activity in AI bilaterally and aMCC, whereas compassion training enhanced activity in the ventral striatum and medial orbitofrontal cortex (mOFC; Klimecki et al., 2013c).

### NEUROMODULATORY BACKDROP

#### Innate immune system

Research from multiple domains supports the idea that empathy and compassionate behavior are diminished by both acute and chronic states of social disconnection. For example, experimental induction of social exclusion is linked to a reduction in empathy and less subsequent prosocial behavior toward others (DeWall and Baumeister, 2006; Twenge et al., 2007). A related body of literature reports a consistent negative relationship between empathy and depression (Cusi et al., 2011). Interestingly, psychoneuroimmunologists have proposed that chronic social isolation biases an individual’s immune system toward the fast-acting innate immune response, characterized by deleterious pro-inflammatory signaling (Cole, 2009). In other studies, enhanced signaling in the innate immune system has been shown to further increase feelings of isolation and enhance amygdala responses to threatening social stimuli (Inagaki et al., 2012), as well as depression (Musselman et al., 2001). Taken together, these studies reveal a powerful cycle whereby isolation and depression enhance inflammation, which then further enhance subjective isolation and decrease empathy and compassion. The optimistic outlook on such a negative cycle is that compassion practices may present an equally powerful intervention that targets the cycle at multiple sites by augmenting both subjective feelings of social connectivity and the biological systems that support it (Pace et al., 2009, 2013). If this is true, then we would hypothesize that decreases in inflammation (e.g., pro-inflammatory cytokines) would mediate changes in social emotions and behavior and related neural functioning.

Desbordes et al. (2012) longitudinal investigation of CBCT in adults naïve to meditation found that, for individuals randomized to compassion meditation but not those randomized to attention meditation, meditation practice time predicted increased amygdala activation in response to compassion-inducing stimuli, though the effect was only marginally statistically significant. Importantly, the increased amygdala activation was associated with reduced levels of depression (Desbordes et al., 2012). This finding is consistent with studies reporting that CBCT reduces inflammatory biomarkers both at rest and in response to psychosocial stress (Pace et al., 2009, 2013), and with other studies showing compassion-based practices lead to decreased depression (Gilbert and Procter, 2006), and supports the idea that one active ingredient in compassion practices is the amelioration of depression and attendant activity of the innate immune response, essentially unmasking the underlying empathy and compassion that were impaired by the individual’s own suffering.

#### Oxytocin

A rapidly burgeoning literature suggests that the oxytocin (OT) system plays an important role in empathy. Research on OT most recently points to a complex, but generally supportive role for OT in the generation of social emotions and behaviors such as trust, empathy, cooperation, social attention, eye gaze, as well as augmentation of the vagal system and dampening of the innate immune and sympathetic response to psychosocial stress [reviewed in Carter (2014)]. Taken together, these findings suggest that the OT system may be involved in mediating the effects of meditation on prosocial emotions and behavior. Moreover, oxytocin’s role as a widely acting neuromodulator (Carter, 2014) might provide a parsimonious explanation for the multitude of effects of kindness-based meditation on both stress physiology and social cognition. However, to the best of our knowledge there is no current evidence that kindness-based meditation alters the OT system. This may be attributed to the fact that central nervous system levels of OT are notoriously difficult to assay and to the potential limitations of plasma measures, which may not accurately reflect OT levels affecting the brain and behavior (Kagerbauer et al., 2013). Beyond circulating levels of OT, the impact of oxytocin on social cognition will also depend on the brain’s sensitivity to it as reflected in receptor density (Insel, 1990), and compassion meditation may up-regulate OT receptors. Unfortunately, a method for directly assessing this possibility *in vivo* does not currently exist, but another possibility, both intriguing and tractable for investigation, is that individual differences in OT receptor polymorphisms, such as those with known relationships with empathy (Rodrigues et al., 2009), may moderate the effects of compassion meditation.

In summary (**Table 2**), the model presented here proposes that empathy is composed of basic attentional, perceptual and motor simulation processes, simulation of another’s affective body state, and slower and higher-level perspective-taking. These components are modulated by emotion regulation and self/other discrimination, and when infused with a motivational component, may become a compassionate response. At all levels in the process, neural systems are influenced by oxytocin and the pro-inflammatory immune system. Kindness-based meditation practices may influence each of these neural systems; however, to date the most consistent evidence supports the idea that LKM enhances the neural systems important for emotion regulation (dlPFC: Weng et al., 2013; vagal tone: Kok et al., 2013) and reward (VTA and mOFC: Klimecki et al., 2013b,c), whereas CBCT affects the perceptual/motor and cognitive processes (Desbordes et al., 2012; Mascaro et al., 2012), perhaps in part by modulating inflammation (Pace et al., 2009, 2013).

**Table 2:** Synopsis of model presented here.

Model	Neural systems involved
Perceptual/motor	Amygdala* (↑) Inferior frontal gyrus*
Affective	Anterior insula Anterior cingulate cortex
Cognitive	Dorsomedial PFC* Temporoparietal junction
Emotion regulation	Amygdala (↑* or ↓) Dorsolateral PFC* Vagus nerve*
Self/other distinction	Temporoparietal junction
**Neuromodulatory backdrop**	
Oxytocin system (↑)	
Pro-inflammatory immune system (↓)*	
**Compassion**	VTA*
	Medial OFC*
	Septal nuclei

*Asterisk indicate regions of the brain that have been shown to be affected by loving-kindness or compassion meditation training.*

## BEST PRACTICES AND FUTURE DIRECTIONS

As has been well-documented (Ospina, 2008; Sedlmeier et al., 2012), the major limitation to identifying mechanisms of action of meditation, including kindness-based practices (Galante et al., 2014), relates to general design weaknesses that, while not unique to meditation research, are arguably especially problematic for it. First, there has been a frequent lack of appropriate comparator groups against which to measure the effects of any given style of meditation, and major confounds such as self-selection and non-specific effects of meditation training have often been left unaddressed. Second, we echo others’ appeals for ecologically valid, objective and implicit assessments of empathy and compassion (Zaki and Ochsner, 2012), and believe that this is especially crucial for examining potential effects of kindness-based meditation practices given the likelihood that demand characteristics and practitioner social desirability render self-report assessments less than optimally reliable (for example, see Hutcherson et al., 2008). Third, researchers in the field of contemplative research, who are often personally committed to the practice of meditation, should be especially mindful to guard against the file drawer effect, or worse, the tendency to under-report findings that would paint meditation in a less positive light. The potential hazards of a research bias in meditation have been illuminated by the work of Willoughby Britton and colleagues (Rocha, 2014), and is supported by accounts in traditional contemplative literature. For example, with respect to compassion practices, Wallace notes that although the long-term effects of compassion are positive, it may be superficially unsettling or upsetting at times (Wallace, 2001). Similarly, the Buddhist scholar Dreyfus (2002, p. 43) has written of beginning bodhisattvas who “are often described as being overwhelmed by compassion. They can be deeply moved by compassion and sometimes cry.” It is likely that similar difficulties may be revealed in studies of western practitioners embarking on compassion meditation, and if so, future research can examine the depth of grief and whether it is simply a necessary obstacle for the beginner to overcome, or rather is integral and motivational, in a sense vital for future outcomes.

It is our hope that the model proposed here will contribute to an ongoing discussion of how best to design, implement and interpret theoretically driven research on compassion and loving-kindness meditation. We suggest that for the field to continue moving forward it will be important to move beyond unitary, single-level outcome measures and rather to employ both peripheral and neural biomeasures, as well as socio-cognitive and behavioral outcome measures that allow testing of mechanistic models within an explicitly defined theoretical framework. Moreover, it will be important for the field to welcome the reporting of negative findings with the understanding that meditation may not be of benefit for all people in all times and places and is unlikely to be a panacea for the many physical and emotional problems that plague the modern world.

The model proposed here also reveals several outstanding questions within the field of social cognitive neuroscience that may be addressed within studies of meditation. Most obvious, this model suggests a dynamic progression of neural processes, but the timing and interrelationships between these dynamic processes remains unclear. A previous study used functional connectivity and causality modeling to determine the interaction between motor simulation in the inferior frontal gyrus and affective simulation in the AI while viewing emotional facial expressions (Jabbi and Keysers, 2008), and similar methodologies could be used to determine the role and relative timing of emotion regulation and self/other distinctions in the dynamic interplay between empathy and compassion. In addition, there is a debate arising within social cognitive neuroscience (Decety and Cowell, 2014) as well as popularized science journalism (Bloom, 2014) regarding the necessity of empathy for compassion, prosocial behavior, and morality, and investigating the outcomes of training the neural systems supporting these discrete aspects of cognition and behavior may be relevant to the discussion. For example, investigations of kindness-based meditation may uncover neural systems that have been up to this point underappreciated for empathy, such as those that underlie the courage or conviction to maintain compassion even when it conflicts with social norms or authority (Bègue et al., 2014).

In addition to theory driven research, we see several under-researched but important questions in the field of meditation research in general, and in compassion and loving-kindness meditation more specifically. As hinted above, the possibility that the effects of meditation practice are not linear, and rather, contain periods of ebb, flow, and even setback during which positive outcomes are less evident remains an underexplored, but crucial topic for basic scientists and clinicians, alike. In addition, research on kindness-based contemplative practices lends itself to the investigation of the ways in which context and meaning impact outcomes. Distinctly different modes of inquiry currently investigate these meditation practices: for individual well-being and therapeutic outcomes on the one hand (e.g., Braehler et al., 2013), and for enhanced social cognitive acuity and prosociality on the other (e.g., Klimecki et al., 2013b). It remains possible that these diverse contexts produce differential subject demand characteristics or otherwise influence outcomes. Similarly, research on mindfulness has benefited from the attention paid to the intentions of the practitioner (Shapiro et al., 2006), and one study has shown that Vipassana practitioner’s goals impacted the outcome of their practice (Shapiro, 1992). Interestingly, our own research with compassion meditation is not consistent with the findings from Vipassana and mindfulness (Mascaro, 2011), as the effects of CBCT were not moderated by practitioner goals, and it may be that practitioner intentions and goals are more influential for particular contemplative practices.

Finally, it would seem obvious that kindness-based contemplative practices might be optimally useful for enhancing empathy and compassion in populations that may stand to benefit most, such as those with psychopathologies typified by empathy deficits (autism, as the most obvious example) or children with as yet under-developed empathic abilities. While this may certainly be the case, it will also be important to simultaneously investigate the real possibility that CM and LKM will be most difficult or least resonant for those that may benefit the most (Rockliff et al., 2008; Mascaro et al., 2014). One potentially fruitful line of research would investigate whether populations that have difficulty adopting a meditation practice are aided by pharmaceutical interventions, such as pre-treatment with oxytocin, that might make the practices more accessible or effective.

Taken together, the studies reviewed here support the idea that compassion and loving-kindness meditation practices alter neural systems thought to be important for empathy and compassion. Intriguingly, the pattern of results, though admittedly incomplete, hints at differential effects of affective and cognitively based practices. On the one hand, LKM appears to target the neural systems important for emotion regulation and reward, whereas CBCT may target the perceptual/motor and cognitive processes. Future work will reveal whether this pattern is indicative of genuine underlying differences in mechanisms of action of the practices, and if so, what such differential mechanisms mean for the behavior and well-being of the practitioner.

## Conflict of Interest Statement

Dr. Charles L. Raison serves on the advisory board for Pamlab and Otsuka-Lundbeck and is a speaker for Pamlab and Sunovion.
